# Higher fasting C-peptide is associated with post-stroke depression: a multicenter prospective cohort study

**DOI:** 10.1186/s12883-021-02413-3

**Published:** 2021-10-04

**Authors:** Yanyan Wang, Wenzhe Sun, Jinfeng Miao, Xiuli Qiu, Yan Lan, Chensheng Pan, Guo Li, Xin Zhao, Zhou Zhu, Suiqiang Zhu

**Affiliations:** Department of Neurology, Tongji Hospital, Tongji Medical College, Huazhong University of Science and Technology, 1095 Jiefang Avenue, Wuhan, 430030 Hubei China

**Keywords:** Fasting C-peptide, Post-stroke depression, Acute ischemic-stroke

## Abstract

**Background:**

Fasting C-peptide (FCP) has been shown to play an important role in the pathophysiology of mood disorders including depression and schizophrenia, but it is unknown whether it also predicts post-stroke depression (PSD). This study examined the association between FCP and PSD at 6 months after acute ischemic-stroke onset among Chinese subjects.

**Methods:**

A total of 656 stroke patients were consecutively recruited from three hospitals of Wuhan city, Hubei province. Clinical and laboratory data were collected on admission. PSD status was evaluated by DSM-V criteria and 17-item Hamilton Rating Scale for Depression (HAMD-17) at 6 months after acute ischemic stroke. The χ2-test, Mann-Whitney U-test, and t-test were used to check for statistical significance. Multivariate logistic regression model was used to explore independent predictor of PSD.

**Results:**

In the univariate analysis, significant differences were found between the PSD and non-PSD groups in terms of FCP level (*p* = 0.009). After multivariate adjustments, FCP remained a significant independent predictor of PSD, with an adjusted odds ratio of 1.179 (95%CI: 1.040–1.337, *p* = 0.010).

**Conclusions:**

Higher FCP levels on admission were found to be associated with PSD at 6 months after acute ischemic-stroke onset. For stroke patients, doctors should pay attention to the baseline FCP for screening high-risk PSD in clinical practice.

## Background

Post-stroke depression is the most common and serious affective disorder after stroke, affecting approximately one-third of stroke patients [1–3]. It is generally known that PSD is linked with higher stroke morbidity, mortality, and recurrence, exerts negative effects on the quality of life of stroke survivors as well as brings heavy burden to caregivers [4, 5]. Although the significance of PSD has been well documented and there are validated screening tools for PSD, a body of PSD patients cannot be diagnosed and treated promptly [6]. One reason is that there are no reliable objective biomarkers for diagnosing and predicting PSD.

So far, the pathogenesis of PSD remains unclear [7]. Previous studies have demonstrated that PSD is associated with an increase in the serum levels of some inflammatory cytokines, such as interleukins IL-1β, IL-6, IL-18, intracellular adhesion molecule 1, tumor necrosis factor-α(TNF-α) and c-reactive protein (CRP) [8–10]. Other molecular markers, including brain-derived neurotrophic factor (BDNF) [11], homocysteine, triiodothyronine [12], uric acid [13, 14], total bilirubin [15] and leptin [16] have also been associated with the development of depression in stroke.

C-peptide, one of the lysis products of proinsulin, is secreted into the circulation in equal amounts to insulin [17]. Compared with insulin, C-peptide is more stable because it is less easily destroyed by the liver [18] and has a longer half-life [19]. Therefore, measuring C-peptide levels can better reflect the function of islet β cell synthesis and the secretion of endogenous insulin, which is widely used in clinical practice. Initially, C-peptide was considered as an inactive molecule. However, currently it is well established that this peptide has significant biological functions, which actually has both anti-inflammatory and pro-inflammatory effects in the body [20–22].

The role of fasting C-peptide level in mood disorders in non-stroke subjects has been explored, with lower fasting C-peptide level was found to be associated with depression [16]. Similarly, Jong et al. [23] found a negative correlation between plasma C-peptide and the self-administered Beck Depression Inventory score in patients with maintenance hemodialysis. Besides, increased concentration of C-peptide was found in schizophrenia patients [24]. To date, however, no study has examined the relationship between fasting C-peptide level and PSD. The lack of data in this field provided the impetus for the study reported herein.

## Methods

### Subjects

**Fig. 1:**
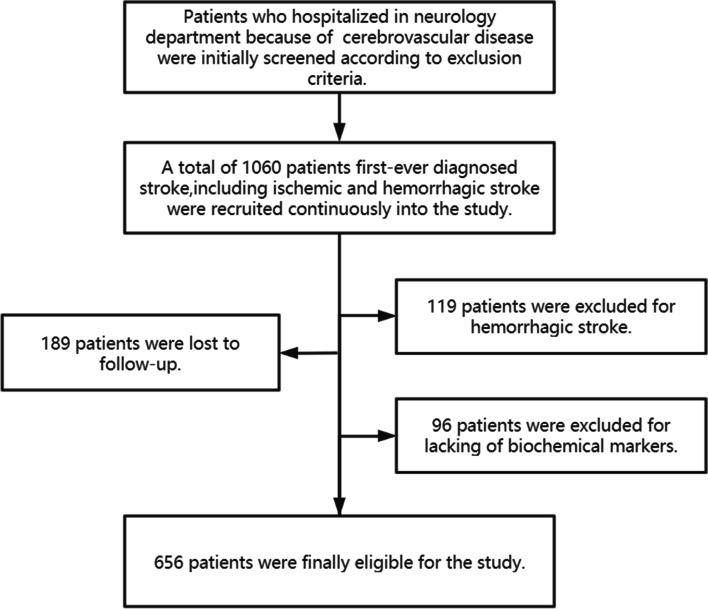
The enrollment flow chart of this study

As a multicenter prospective cohort study, our research was conducted at three independent hospitals (Tongji Hospital, Wuhan First Hospital and Wuhan Central Hospital) between August 2018 and June 2019. Inclusion and exclusion criteria for enrollment of patients has been previously reported [14]. A total of 1060 patients first-ever diagnosed stroke, including ischemic as well as hemorrhagic stroke, were consecutively enrolled and 656 patients were finally eligible for the research (Fig. 1).

This study was approved by the ethics committee of Tongji Medical College, Huazhong University of Science and Technology (Approved No. of ethic committee: TJ-IRB20171108). According to the Declaration of Helsinki, all subjects signed an informed consent form.

### Data collection

A standardized questionnaire was used Upon admission to collect detailed information about each patient’s demographic and medical history, including age, gender, height, weight, sleep time, educational level, smoking history, drinking history, hypertension, diabetes mellitus (DM), hyperlipemia and history of coronary heart disease (CHD). Hospitalization days were also included into the variables, which derived from the patient’s discharge records.

Blood samples were obtained from the medial cubital vein in the sitting position at fasting in the early morning. We measured fasting C-peptide (FCP), adrenocorticotropic hormone (ACTH), c-reactive protein (CRP), cortisol, total WBC count (WBC), neutrophil count (NEU), lymphocyte count (LYM), monocyte count (MONO), interleukin (IL-1β, IL-6, IL-10, IL-18), tumor necrosis factor (TNF-α), and interferon (IFN-γ).

The National Institutes of Health Stroke Scale (NIHSS), Barthel index (BI), Modified Rankin scale (mRS), Mini-Mental State Examination (MMSE), Conner-Davidson Resilience Scale (CD-RISC) and Neuroticism scale (subscale of Eysenck Personality Questionnaire) were evaluated within 3 days of the patient’s admission. The 17-item Hamilton Rating Scale for Depression (HAMD-17) had been proved to have good reliability and validity in Chinese population [25], which therefore was used to measure the severity of depressive symptoms at 6 months after ischemic stroke onset.

Participants who met DSM-V diagnostic criteria (depression caused by other medical conditions) along with HAMD-17 score > 7 were assigned to depression group (PSD) [14], otherwise, they were assigned to non-depression group (NPSD). The reason why we chose 6 months after ischemic stroke onset for depression assessment is mainly to avoid the period of transient emotional adjustment to the disability caused by the stroke.

### Statistical analysis

Data analysis were performed using the Statistical Program for Social Sciences (SPSS) statistical software (version 25, Chicago, IL, USA). Categorical variables were expressed as percentages and were compared using χ2-test. Continuous variables were presented as medians [interquartile range (IQR)] or means ± standard deviation (S.D.) depending on the normal or nonnormal distribution of data by Kolmogorov-Smirnov test and were compared using t-test or Mann–Whitney U-test (when continuous variables had skewed distributions) [14]. Every variable was analyzed by univariate analysis to cover all potentially important predictors. Variables with *P* < 0.05 were considered statistically significant.

Subsequently, baseline variables that have been proved to be clinically relevant or that showed a univariate relationship with PSD at 6 months after stroke were entered into multivariate logistic regression model. Variables for inclusion were carefully chosen, given the number of events available, to ensure parsimony of the final model. Collinearity of variables that entered the multivariate logistic regression analysis was assessed by variation inflation factors (< 5 being considered nonsignificant) and tolerance (> 0.2 being considered nonsignificant). The multivariable logistic regression using a backward stepwise method with input of variables if *p*-value < 0.05 and backward elimination if *p*-value > 0.05. All *p*-values were two-sided, *p* < 0.05 was considered statistically significant.

## Results

### Baseline characteristics

In total, 656 patients with acute ischemic stroke were consecutively recruited in this study. Of all these potential subjects, 236 (36%) finally developed PSD at 6 months after ischemic stroke onset. Table 1 shows a comparison of baseline information between the NPSD and PSD groups. The PSD and non-PSD groups did not differ in terms of age or BMI, but the PSD group had a higher proportion of female (*p* = 0.013), lower education level (*p* = 0.004), longer hospitalized time (*p* = 0.002), shorter sleep time (*p* = 0.002), higher NIHSS score (*p* < 0.001), lower BI score (*p* < 0.001), higher m RS score (*p* = 0.002), lower CD-RISC score (*p* = 0.008) and higher neuroticism score (*p* = 0.027). As for serum biochemicals, the PSD group showed higher level of FCP (*p* = 0.009), higher white blood cell count (*p* = 0.037), higher neutrophil count (*p* = 0.018) and higher monocyte count (*p* = 0.006).

**Table 1:** Demographic and clinical characteristics of patients without and with PSD

Parameter	NPSD(*N* = 420) 64%	PSD(*N* = 236) 36%	*P*
**Demographic information**			
Age, median (IQR)	58(51, 66)	60(52, 67)	0.143
Females, n (%)	82(19.50)	66(28.00)	0.013*
BMI, median (IQR)	24.45(22.50, 26.90)	24.20(22.50,26.73)	0.387
hospitalization days, median (IQR)	10(8, 13)	11(8, 15)	0.002*
Sleep time < 5 h, n (%)	44(10.50)	45(19.1)	0.002*
Education level, n (%)	189(45.00)	79(33.50)	0.004*
Smoking, n (%)	190(45.20)	99(41.90)	0.415
Drinking, n (%)	195(46.40)	93(39.40)	0.082
Hypertension, n (%)	249(59.30)	136(57.60)	0.679
Diabetes mellitus, n(%)	120(28.60)	67(28.40)	0.961
Hyperlipemia, n (%)	106(25.20)	73(30.90)	0.116
CHD, n (%)	43(10.20)	26(10.50)	0.755
**Serum biochemicals**			
FCP, median (IQR)	1.82(1.14, 2, 49)	2.00(1.38, 2.70)	0.009*
ACTH, median (IQR)	30.90(16.64, 44.43)	32.40(18.61,47.95)	0.229
CRP, median (IQR)	1.80(0.80, 5.48)	2.50(0.93, 5.89)	0.087
Cortical, median (IQR)	13.00(10.33, 15.70)	13.50(10.93,16.20)	0.077
WBC, median (IQR)	6.57(5.39, 8.08)	6.89(5.66, 8.50)	0.037*
NEU, median (IQR)	4.11(3.25, 5.46)	4.36(3.47, 5.80)	0.018*
MONO, median (IQR)	0.50(0.39, 0.62)	0.53(0.44, 0.67)	0.006*
LYM, median (IQR)	1.58(1.27, 2.09)	1.65(1.23, 1.99)	0.904
IL.1β, median (IQR)	66.47(28.05, 165.07)	63.60(26.05,167.02)	0.894
IL.6, median (IQR)	6.00(2.76,9.54)	6.00(2.60,10.06)	0.242
IL.10, median (IQR)	9.13(2.89,22.96)	7.78(2.57,19.73)	0.286
IL.18, median (IQR)	2158.84(1012.72,4753.75)	1896.82(954.22,4913.12)	0.439
TNF-α, median (IQR)	37.11(21.85,57.62)	41.42(23.20,59.97)	0.260
IFN-γ, median (IQR)	4.33(1.50,8.27)	5.10(2.00,9.72)	0.063
**Clinical characteristics**			
NIHSS, median (IQR)	2(1,5)	3(2,7)	<0.001*
BI, median (IQR)	95(65,100)	80(40,100)	<0.001*
MRS, median (IQR)	2(1,3)	2(1,4)	0.002*
CD-RISC, median (IQR)	65(54,77)	61(50,73)	0.008*
N, median (IQR)	8(4,11)	9(6,13)	0.027*

*PSD* Post-stroke depression, *IQR* Interquartile range, *BMI* Body mass index, *CHD* Coronary heart disease, *FCP* Fasting C-peptide, *ACTH* Adrenocorticotropic hormone, *CRP* C-reactive protein, *WBC* White blood count, *NEU* Neutrophil count, *LYM* Lymphocyte count, *MONO* Monocyte count, *IL* Interleukin, *TNF-α* Tumor necrosis factor-α, *IFN* Interferon, *NIHSS* The National Institutes of Health Stroke Scale, *BI* Barthel Index, *m RS* Modified Rankin Scale, *CD-RISC* Conner-Davidson Resilience Scale, *N* Neuroticism

*Statistically significant at *p* < 0.05 level, two-sided

### Independent predictors of PSD

Baseline variables that showed p<0.05 in univariate analysis (Table 1) or that have been proved to be clinically relevant were finally included in multivariate logistic regression model. Variance Inflation Factor (VIF) was used to check for multicollinearity among each variable and no significant statistical collinearity was observed for these variables. After adjusting by potential confounders, educational level (OR = 0.677,95%CI:0.479–0.958, *p* = 0.028), sleep time (OR = 1.998, 95%CI: 1.250–3.192, *p* = 0.004), hospitalization days (OR = 1.029,95%CI: 1.001–1.059, *p* = 0.042), FCP (OR = 1.179,95%CI: 1.040–1.337, *p* = 0.010), NIHSS (OR = 1.100, 95% CI: 1.045–1.157, *p* = 0.000) and CD-RISC (OR = 0.987, 95% CI: 0.977–0.997, *p* = 0.011) remained independently and significantly related with PSD at 6 months after the onset of ischemic stroke (Table 2).

**Table 2:** Multivariate logistic regression model for PSD

Parameter	β	SE	*P*	OR (95%CI)
Educational level	−0.389	0.177	0.028*	0.677(0.479–0.958)
Sleep time	0.692	0.239	0.004*	1.998(1.250–3.192)
Hospitalization days	0.029	0.014	0.042*	1.029(1.001–1.059)
FCP	0.165	0.064	0.010*	1.179(1.040–1.337)
NIHSS	0.095	0.026	0.000*	1.100(1.045–1.157)
CD-RISC	−0.013	0.005	0.011*	0.987(0.977–0.997)

Variables for inclusion:age, gender, hospitalization days, educational level, sleep time, hypertension, diabetes mellitus, hyperlipemia, CHD, FCP, ACTH, cortical, CRP, WBC, NEU, LYM, MONO, NIHSS, MRS, BI, CD-RISC, N

*PSD* Post-stroke depression, *FCP* Fasting C-peptide, *NIHSS* National Institutes of Health stroke scale, *CD-RISC* Conner-Davidson Resilience Scale, *SE* Standard Error, *OR* Odds Ratio, *CI* Confidence Interval

*Statistically significant at *p* < 0.05 level, two-sided

## Discussions

In this multicenter prospective cohort study, 36% of acute ischemic stroke patients were diagnosed as PSD at 6 months after stroke onset, which is consistent with the results of previous researches [2, 26]. Our study suggested that higher level of C-peptide, higher NIHSS score, longer hospital stay and shorter sleep time were risk factors for PSD. Furthermore, higher CD-RISC score and higher educational level were protective factors lowering the risk of PSD.

For most people, when it comes to C-peptide, the first thing that comes to mind is diabetes. However, a growing body of research suggests that C-peptide also plays an important role in the occurrence and development of other diseases. Several studies have shown positive correlation between C-peptide levels and the incidence of angio-cardiopathy, such as coronary artery disease and myocardial infarction [27–29]. In addition, studies on the relationship between C-peptide and depression have been increasing in recent years. Jong et al. found serum levels of C-peptide was negatively correlated with symptoms of depression in patients on maintenance hemodialysis [23]. Similarly, Kudo et al. found lower C-peptide level was associated with depression [16]. Contrary to their conclusions, our data suggests that higher fasting C-peptide level is related to depression in stroke patients.

Higher C-peptide levels may impact post-stroke depression indirectly through many mechanisms. The most plausible explanation is that high levels of C-peptide reflect insulin resistance and disrupted glucose metabolism, which contributes to depression. It is well known that chronic impairment of glucose metabolism is closely linked to depressive disorders [30] and the prevalence of depression is significantly higher in diabetes mellitus patients [31]. Depressive symptoms often appear at the prediabetes stage characterized by insulin resistance [32]. To a certain degree, as insulin is involved in depression, prevention and treatment insulin resistance may improve symptoms of depression. Considering there are many similarities between the pathogenesis of depression and post-stroke depression, it is reasonable to deem that high C-peptide levels as well as insulin resistance are associated with post-stroke depression. Another reasonable explanation is that C-peptide indirectly contributes to depression through its role in promoting atherosclerosis. A previous study has demonstrated that the deposit C-peptide on intima layer of the vessels promotes infiltration of monocytes/macrophages and lymphocytes CD4+, with impacts on the atherogenesis process [33–35]. Cerebrovascular atherosclerosis causes arterial stenosis, which leading to hemodynamic disturbance as well as low perfusion. Studies have shown that the hemodynamics changes of the middle cerebral artery are linked to the onset of depression in the elderly [36]. Besides, animal studies have also shown that depression-like behavior in rats was related to cerebral hypoperfusion [37]. Hence, cerebrovascular atherosclerosis could be a partial explanation for the link between C-peptide level and PSD. In summary, the underlying mechanism between C-peptide and post-stroke depression is not fully understood and needs further study.

The NIHSS score is regularly used as the measure of stroke severity upon admission. Consistent with the previous studies [38], we demonstrated that the NIHSS score serves as a strong risk factor for depression after acute ischemic stroke. Patients with high NIHSS scores tended to have more serious physical disability and therefore need to be stay in hospital more days receiving rehabilitation treatment. To a certain extent, longer hospital stay indicates higher medical costs, which brings serious psychological burden to patients and thus promotes the occurrence of depression.

The link between depression and poor sleep quality has been shown in numerous studies [39–42] . As many as 90% of depressed patients experience poor sleep. Depression can lead to sleep disturbances and, conversely, poor quality nighttime sleep can inhibit daytime function and produce depression. A vicious cycle may unfortunately exist between sleep and depression.

Our study suggests that higher education levels is protective against post-stroke depression. This is because, compared with patients with lower education levels, patients with higher education levels tend to have better self-regulation ability and more social resources to cope with negative life events [43].

Psychological resilience is defined broadly as positive emotional and/or behavioral adaptation to adversity. The higher the score of psychological resilience, the stronger the ability to adapt to stress [44, 45]. Stroke patients who can be able to face difficulties with an optimistic attitude are less likely to be depressed, which is consistent with previous studies.

### Limitations

A limitation of this study is that patients with dysarthria, aphasia or other diseases were excluded, and these excluded patients might be suffered from depressive symptoms. This might cause a selection bias, resulting in a lower rate of PSD than actual data. What’s more, the loss rate of follow-up reduced some statistical power of the analyses. Finally, because this was a multicenter study, the accuracy of instruments varied from hospital to hospital.

## Conclusions

Higher level of fasting C-peptide on admission is associated with the risk of PSD at 6 months after acute ischemic-stroke onset. Further investigations are needed to clarify the underlying pathophysiological link between fasting C-peptide level and PSD. For stroke patients, doctors should pay attention to the baseline FCP for screening high-risk PSD in clinical practice.

## Data Availability

The de-identified database used in the current study are available from the corresponding author on reasonable request.

## Abbreviations

FCP: Fasting C-peptide
PSD: Post-stroke depression
HAMD-17: 17-item Hamilton Rating Scale for Depression
TNF-α: Tumor necrosis factor-α
CRP: C-reactive protein
BDNF: Brain-derived neurotrophic factor
CT: Computed tomography
MRI: Magnetic resonance imaging
TIA: Transient ischemic attack
BMI: Body mass index
CHD: Coronary heart disease
ACTH: Adrenocorticotropic hormone
WBC: White blood count
NEU: Neutrophil count
LYM: Lymphocyte count
MONO: Monocyte count
IL: Interleukin
IFN: Interferon
NIHSS: The National Institutes of Health Stroke Scale
BI: Barthel Index
m RS: Modified Rankin Scale
MMSE: Mini-Mental State Examination
CD-RISC: Conner-Davidson Resilience Scale
N: Neuroticism
